# Human Chorionic Gonadotropin and Related Peptides: Candidate Anti-Inflammatory Therapy in Early Stages of Sepsis

**DOI:** 10.3389/fimmu.2021.714177

**Published:** 2021-09-13

**Authors:** Sun Koo Yoo, Syed Faizan Mehdi, Suma Pusapati, Nimisha Mathur, Manasa Anipindi, Bruno Lunenfeld, Barbara Lowell, Huan Yang, Christine Noel Metz, Sawleha Arshi Khan, Derek Leroith, Jesse Roth

**Affiliations:** ^1^The Feinstein Institutes for Medical Research/Northwell Health, Manhasset, NY, United States; ^2^Faculty of Life Sciences, Bar-Ilan University, Ramat Gan, Israel; ^3^Division of Endocrinology, Diabetes & Bone Disease, Icahn School of Medicine at Mt. Sinai, New York, NY, United States

**Keywords:** inflammation, sepsis, cytokine storm, human chorionic gonadotrophic hormone (hCG), anti-inflammatory

## Abstract

Sepsis continues to be a major cause of morbidity, mortality, and post-recovery disability in patients with a wide range of non-infectious and infectious inflammatory disorders, including COVID-19. The clinical onset of sepsis is often marked by the explosive release into the extracellular fluids of a multiplicity of host-derived cytokines and other pro-inflammatory hormone-like messengers from endogenous sources (“cytokine storm”). In patients with sepsis, therapies to counter the pro-inflammatory torrent, even when administered early, typically fall short. The major focus of our proposed essay is to promote pre-clinical studies with hCG (human chorionic gonadotropin) as a potential anti-inflammatory therapy for sepsis.

## Introduction

### Sepsis

Sepsis is a clinical syndrome characterized by physiologic and biochemical abnormalities associated with organ injury caused by dysregulated host responses to infection (and or inflammation) (1). Sepsis is typically associated with multiple organ failure and a high rate of morbidity and mortality (2, 3). The World Health Organization (WHO) reports over 30 million cases in the world every year with approximately 6 million deaths (4). In the United States, 1.7 million adults develop sepsis each year associated with 270,000 deaths (5). Death rates continue to increase (6). Hospitalizations are often long, often with slow and incomplete recoveries. Prolonged or permanent disability and death are also common.

### Emergence of Sepsis

In the healthy individual, pro-inflammatory molecules are roughly balanced by anti-inflammatory elements. In sepsis, multiple intercellular communication pathways are disturbed leading to elevated and sustained pro-inflammatory agents, both helping and harming the host. In about two-thirds of patients with sepsis, infection will be recognized as a dominant cause (7). In the other third of the patients, no infection is detected; the sepsis is ascribed to one or more non-infectious inflammatory disorders e.g., pancreatitis, burns, severe trauma, head injury, or ischemia-reperfusion (1, 8).

Early in the course of sepsis when infection is uncertain, clinicians typically (i) culture multiple sites, (ii) immediately initiate treatment with multiple broad spectrum antibiotics (iii) while awaiting culture results. Often antibiotic treatment increases the *in vivo* dominance of pro-inflammatory messenger molecules.

A major expected but undesirable consequence of broad-spectrum antibiotic therapy is a reduction in the host’s native microbes, especially those of the intestines. This reduction includes their number, range of species (i.e., diversity), and their production of molecules of metabolism and intercellular communication. Disruption of the microbiota can significantly alter the host’s immune system (9). Under normal circumstances, the intestinal microbes produce more anti-inflammatory agents relative to pro-inflammatory messenger molecules maintaining peaceful balance (“pax intestinalis”) (1). With the use of antibiotics, the patient’s endogenous microbes that usually supply anti-inflammatory messengers are markedly diminished, further promoting the pro-inflammatory dominance.

## Overall Vision

**Figure 1:**
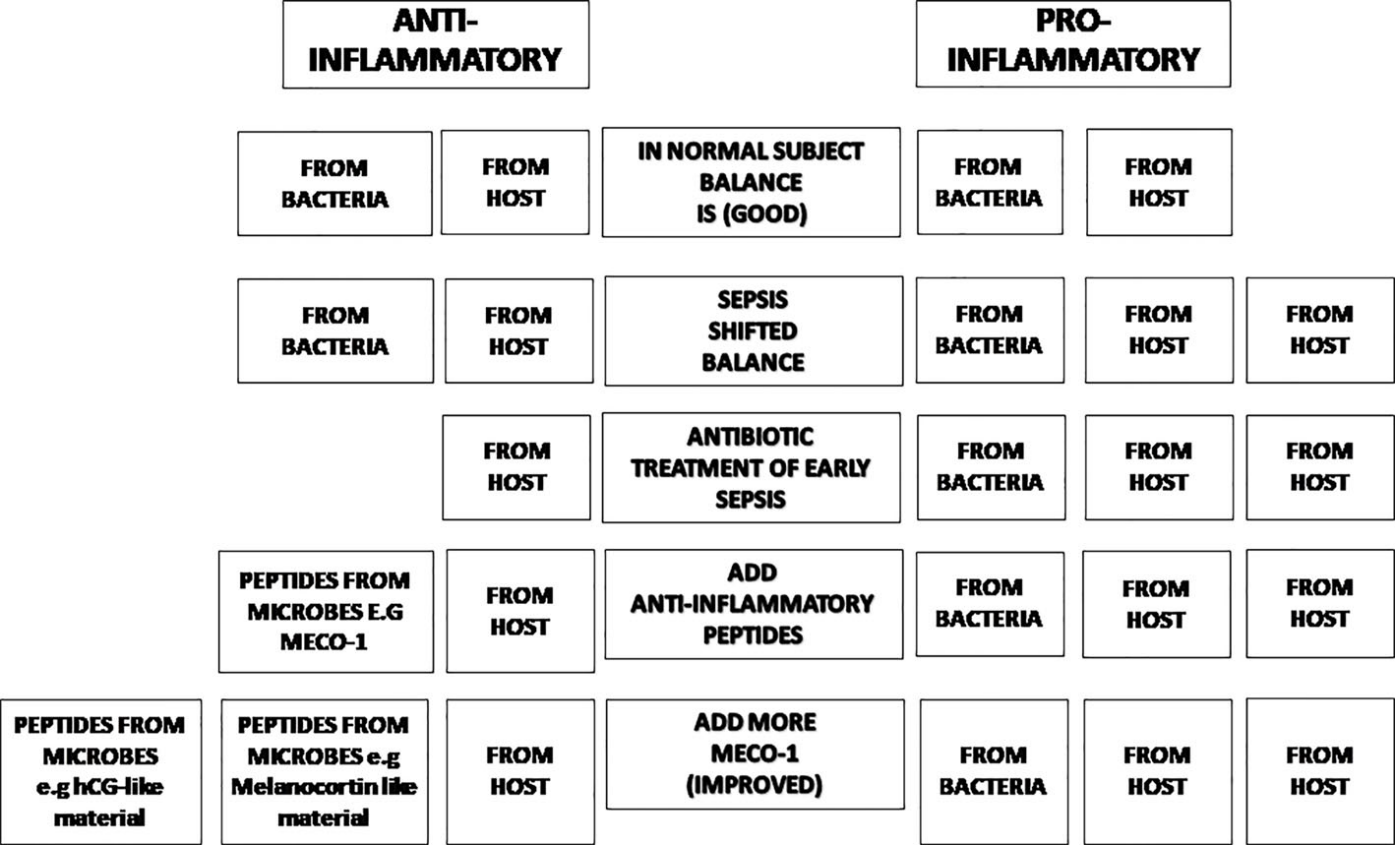
The balance of inflammatory status. In normal subjects, the inflammatory status is balanced (Line 1). Once there is inflammation in the subject, the balance will shift in favor of pro-inflammatory effectors (Line 2). Antibiotic treatment moves the trend to more pro-inflammatory status due to its bactericidal effect (losing anti-inflammatory agents from microbes) (Line 3). Anti-inflammatory peptides from microbes (such as MECO-1 or hCG-like material) will help restore the balance (Line 4 & Line 5).

Our long-range proposal is to provide anti-inflammatory peptides to patients with sepsis as soon as they are started on antibiotics to promote the balance between pro-inflammatory and anti-inflammatory messenger molecules to improve outcomes. We propose to use well-studied hormones and their analogs, individually and in unison with mice treated with broad-spectrum antibiotics, likely to suffer from sepsis. Our menu of experiments will include microbe-induced sepsis with one organism, (e.g., pneumococcus), and multiple organisms (e.g., cecal ligation and puncture). We also plan to study sterile (microbe-free) sepsis (e.g., post endotoxin or post recovery from sepsis) (**Figure 1**).

This manuscript will catalog data that leads us to hypothesize that human chorionic gonadotropin (hCG) and its relatives from mammalian and microbial sources may provide benefits when administered early in sepsis. One significant advantage that will permit speedy progress with hCG is the vast experience with its use in laboratory animals and humans, as well as its long-standing approval by the FDA for multiple uses in humans.

## History of Human Chorionic Gonadotropin

hCG was discovered after decades of extensive research by many pioneers. In 1920, Hirose demonstrated that placental extracts stimulated ovulation in rabbits and guinea pigs (10, 11)(see **Table 1**). Seven years later, Aschheim and Zondek reported that the urine of pregnant women contained a substance that resembled an anterior pituitary lobe hormone. This substance first appears in significant amounts in the urine shortly after fertilization (15). When this substance was injected into immature mice, it induced precocious sexual maturity i.e., follicular maturation, hemorrhages into follicles and luteinization of follicles in their ovaries (12–17). This discovery eventually led to the development of a rapid urine test for pregnancy (15, 18–21). In 1929, Zondek discovered that the pituitary gland secreted two hormones that stimulated gonads: Prolan A and Prolan B which became follicle stimulating hormone (FSH) and luteinizing hormone (LH) respectively. Fourteen years later in 1943, Seegar-Jones and colleagues demonstrated that the substance isolated from urine of pregnant women was actually produced by giant syncytiotrophoblast cells of the placenta, not by the pituitary gland (12, 14).

**Table 1:** History of hCG (12–14).

1920	Hirose demonstrated that placental extracts stimulated ovulation in rabbits and guinea pigs
1927	Aschheim and Zondek identified a substance in urine from pregnant women that stimulated ovary
1931	First commercially available hCG extract, (Pregnon) was introduced
1932	Pregnon name changed to Pregnyl
1940	Purified preparations of hCG from urine became available
1943	hCG was proven to be released by placenta, not by pituitary
2000	Recombinant hCG approved for use (only for women according to FDA)

All alpha subunits have identical 92 amino acid sequences. The β chain differs in sequence and molecular weight.

Abundant research over several decades made it possible to isolate more pure and potent forms of hCG. In 1931, a placental extract for the stimulation of ovaries was made commercially available by Organon with the brand name Pregnon (12, 22). In 1932, the name was changed to Pregnyl to avoid resemblance with another trademark. hCG preparations are still available today under the trade name Pregnyl. At first, biological activity of hCG extracts was calibrated in animal units such as “rat units.” In 1939, the League of Nations introduced the international unit (IU) that was a new global standard unit of hCG, which greatly increased the reproducibility of the purified forms (13, 23, 24). Purified hCG was extracted from urine for the first time in the 1940s (13, 23, 24). Later in 2000, recombinant hCG preparations became available (13). Currently, urinary and recombinant hCG preparations are widely available from several commercial sources (25), as they are commonly used in the management of infertility and prepubertal cryptorchidism, as well as for stimulating testosterone production in hypogonadal men.

## The Glycoprotein Hormone Family

**Figure 2:**
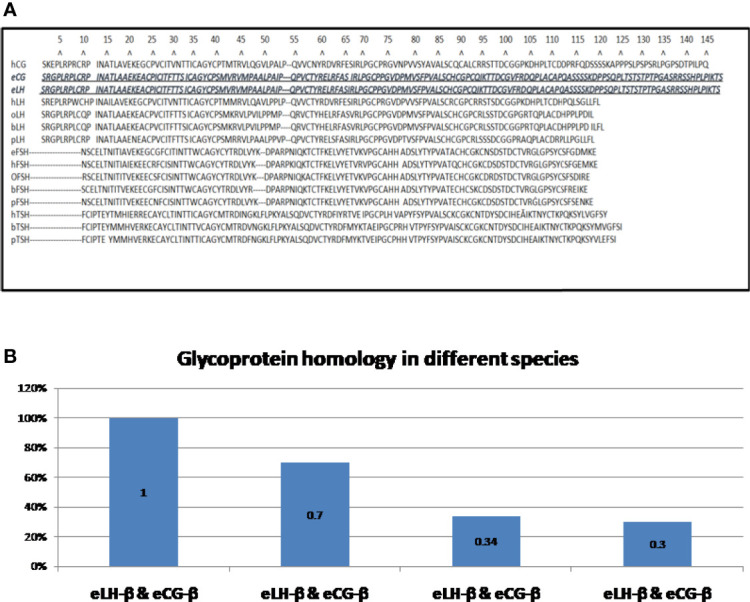
**(A)** Amino acid sequences of the β-subunits of glycoprotein hormones (34): The shortest β-subunit in the family is bovine FSH-β consisting of 111 amino acids; the longest β-subunits in the family are of equine LH-β and equine CG-β consisting of 149 amino acids (equine LH-β & equine CG-β are identical) (26). h = human, e = equine, o = ovine, b = bovine, p = porcine CG = chorionic gonadotrophin; LH = luteinizing hormone; FSH = follicle-stimulating hormone; TSH = thyroid stimulating hormone. **(B)**. Homology of β-subunits of glycoprotein hormones. Exceptionally, the amino sequences of equine LH-β and equine CG-β are identical (26). Human LH-β and human CG-β are about 70% homologous. Ovine LH-β and ovine FSH-β are about 34% homologous. Bovine LH-β and bovine CG-β are about 30% homologous. H, human; e, equine; o, ovine; b, bovine; p, porcine; CG, chorionic gonadotrophin; LH, luteinizing hormone; FSH, follicle-stimulating hormone; TSH, thyroid stimulating hormone.

The glycoprotein hormone family in mammals has four closely related entities, chorionic gonadotropin (CG), luteinizing hormone (LH), follicle stimulating hormone (FSH), and thyroid-stimulating hormone (TSH). CG is mainly produced by placenta while LH, FSH, and TSH are mainly produced by pituitary cells (26). Each glycoprotein hormone consists of one α-subunit and one β-subunit that are non-covalently associated (27). The α-subunits of all four hormones are identical (28); a free unbound α-subunit does not have any known independent biological function (29, 30). The β-subunit of the four hormones give biological specificity to each hormone (31)and share some homology in their amino acid sequences (26); CG and LH both bind to the same receptor known as the luteinizing hormone chorionic gonadotropin (LHCG) receptor. FSH binds to FSH receptor and TSH binds to TSH receptor (28, 32, 33). All three receptors are G-protein-coupled to post-receptor pathways (32). **Figure 2A** shows that the lengths of the β-subunits vary. Also note that human CG and equine CG each have a unique C-terminal addition that makes them the largest molecules in the family (26, 35) (See **Figures 2A, B**).

## Human Chorionic Gonadotropin

Unique among the glycoprotein hormones, hCG is mainly produced by syncytiotrophoblast cells of placenta which are the main source of hCG found in the blood and excreted in the urine (36). In early pregnancy, it contributes to the maintenance of the corpus luteum (37, 38), which in turn provides progesterone that is essential for successful pregnancy progression.

### Structure of hCG

Like the other hormones in this family, hCG is composed of one α-subunit and one β-subunit (39). The α-subunit of hCG contains 92 amino acids with two N-glycosylation sites. It is encoded by a single gene, CGA that is located on chromosome 6q21.1-23 (40). The β-subunit of hCG contains 145 amino acids with two N-glycosylation sites and four O-glycosylation sites. It is encoded by six non-allelic genes (abbreviated CGB) clustered on chromosome 19q13.3 (CGB1, CGB2, CGB3, CGB5, CGB7 and CGB8) (28). The coordination of the six genes and how these six genes lead to the production of one protein are not yet well defined. The α-subunit and β-subunit are extensively intertwined, held together by non-covalent hydrophobic and ionic interactions. The C-terminus of the β-subunit wraps around the α-subunit which is important for subunit assembly. The details of the extensive interface give hints of how α-subunits interact with and associate with the β-subunits of different hormones (31).

### Strength of Binding to the Receptor

In terms of electrostatic charge and strength of binding to receptor, the hCG’s surface electrostatic potential is positive at or near the receptor-binding interface of hCGreceptorand negative on the opposite side. The stronger positive charge yields tighter binding; the less positive charge provides weaker binding. Negatively charged residues in the hCG receptor lower its affinity for binding (31).

### Size and Weight of hCG

**Figure 3:**
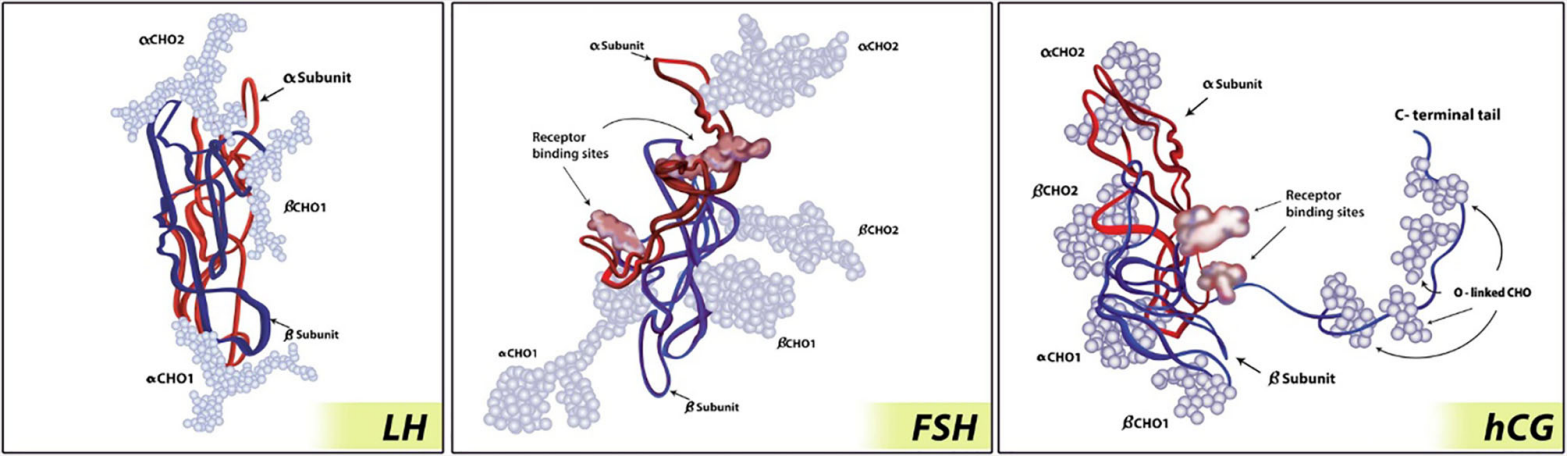
Glycoprotein hormone molecule (73). hCG has the largest size of the glycoprotein hormones due to carboxy-terminal addition. The α-subunits are represented by red strand; the β-subunits are represented by blue strand; carbohydrate chains are represented by light blue balls. Figure from SpringerLink (73) permissible to reuse under a CC-BY 4.0 license.

In terms of size and weight, hCG is the largest and heaviest in the mammalian glycoprotein hormone family (28, 40–45). Its β-subunit has a C-terminus with a 31-amino acid extension as well as four additional carbohydrate moieties (46). Together these make hCG a substantially larger molecule than the other mammalian glycoprotein hormones (31) (**Table 3** and **Figure 3**).

**Table 3:** Human CG *vs* microbial CG (Xanthomonas and Candida Chorionic Gonadotropin).

	Molecular Weight	Size (Base Pairs)
**Human CG**	37 kDa	711 bps
**Xanthomonas CG**	48 kDa	1362 bps
**Candida CG**	68 kDa	––––

In terms of size and weight, microbial CGs are larger than human CG. The molecular weight of Candida CG is 68 kDa compared to 48 kDa of Xanthomonas CG and 37 kDa of human CG. Xanthomonas CG has 1362 base pairs in its sequence that can be converted to 454 amino acids (1362 bp/3 = 454 AAs), and hCG has 711 base pairs that can be converted to 237 amino acid that calculated from the addition of 92 of α-subunit and 145 of β-subunit (28, 46, 47).

### Cells Producing hCG

Cells in the placenta produce nearly all of the hCG. Small amounts of hCG can also be found in human tissues other than placenta e.g., liver, kidney, and lung (48). Unlike placenta, these tissues do not secrete hCG into blood. The function of hCG produced by the non-placental tissue is not known. Typically, non-placental normal human pituitary cells do secrete low levels of hCG into blood during the middle of menstrual cycle (49). It mimics LH actions in the menstrual cycle (50), but the specific function of pituitary hCG is not well understood (48). Multiple primary malignant cells such as those from colon cancer, ovarian cancer, and breast cancer also secrete hCG (46, 51–54). This is considered to be a sign of poor prognosis, possibly because the free β-subunits prevent apoptosis of malignant cells, thereby enhancing the malignant cell growth (50).

## Gonadotropin From Microbes

### Peptides Secreted by Microbes

The search for peptides secreted by microbes similar to mammalian hormones started more than a half century ago. Our lab group reported TSH-like material in Clostridium perfringens as well as insulin-related material in Escherichia coli, and melanocortin-related material in E. coli and other microbes. Other groups found insulin-related materials, somatostatin-like materials, calmodulin and calcitonin (55–57). Several strains of bacteria were found to release neurotensin (58). Recently, we characterized a melanocortin-like peptide secreted from E. coli (MECO-1) that has anti-inflammatory effects (59). MECO-1 is a 33-amino acid peptide released by E. coli that is homologous to the C-terminus of the E. coli elongation factor-G (EF-G); it is similar to alpha melanocyte-stimulating hormone (α-MSH) and adrenocorticotropic hormone (ACTH) in structure and even more so in bioactivities exercised through the melanocortin-1-receptor (MC1R) (59).

**Figure 4:**
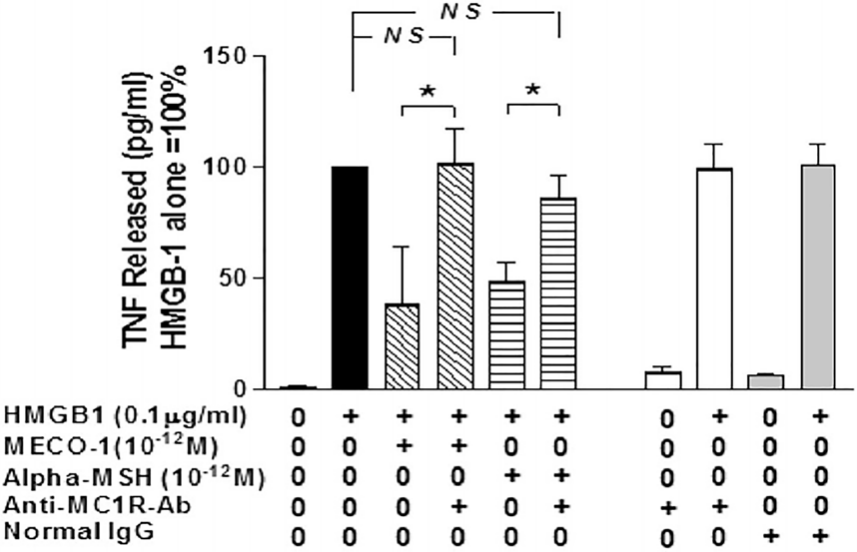
MC1R antibody blocks the inflammatory suppressant effect of MECO-1 and of α-MSH.[Reproduced from (59)]. Murine macrophages-like RAW 264.7 cells were pretreated with anti-MC1R Ab for 10 min prior to addition of HMGB1 with/without MECO-1 or α-MSH at 10-12 M. The TNF content of the cell- free medium was determined by ELISA after 6hr. Figure from npj biofilms (59) permissible to reuse under a CC-BY 4.0 license. (*) refers to statistically significant p value i.e *P* < 0.05. NS means not statistically significant p value i.e. *P* > 0.05.

MECO-1, α-MSH, and ACTH were effective in blunting the release of (i) pro-inflammatory cytokines, (ii) high mobility group box 1 (HMGB1), and (iii) tumor necrosis factor (TNF) from macrophage-like cells activated by exposure to lipopolysaccharide (LPS) or HMGB1. *In vitro*, the anti-inflammatory properties of MECO-1 and α-MSH were abolished by antibodies against (MC1R) and by agouti signal protein, an inverse agonist of MC1R from mammals. *In vivo* MECO-1 showed greater capacity than α-MSH to protect mice from lethal doses of LPS or sepsis induced by cecal ligation and puncture (CLP) (59); possibly MECO-1 has longer survival *in vivo* (**Figure 4**).

### hCG-Related Peptides in Microbes

Initially thought to be produced only in mammals and other chordates, CG-related peptides have been reported in multiple microorganisms e.g. Staphylococcus species, Corynebacterium ulcerans, Eubacterium lentum, Escherichia coli, Stenotrophomonas (Xanthomonas) maltophilia, and Progenitor cryptocides. Some species of Streptococcus, and of Candida express mRNAs and proteins that resemble transcripts and proteins of CG. Some of the microbe-derived peptides have been shown to produce as well as secrete gonadotropin-like peptides (41, 46–49, 51–54, 60–69).

In the 1970s, several anaerobic and aerobic bacteria isolated from patients with a range of malignant tumors (including colon, ovary, breast and lymph node) were found to release hCG-like material when assayed for the β-subunit of hCG (51, 54). hCG-like substances were reported in cancer patients. hCG was also detected in some bacteria and yeast from patients independent of the presence of a tumor (36). hCG-like material was detected not only in microbes that were commonly found in humans such as Staphylococcus epidermidis, S. hominis, S. haemolyticus, and Candida albicans, but also in bacteria that are less common residents of human microbiota (68, 70).

**Figure 5:**
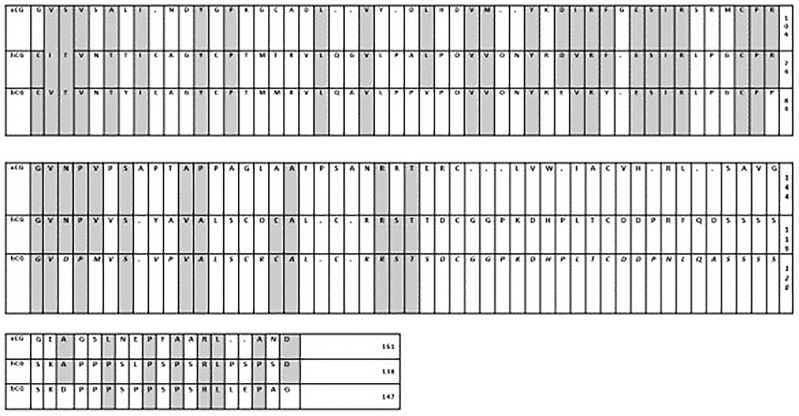
Comparison of alignments of Xanthomonas CG with human CG and baboon CG (46). When Xanthomonas CG was aligned with human CG and baboon CG, there were 46% similarities and 28% identities. xCG, Xanthomonas CG; hCG, human CG; bCG, baboon CG.

Xanthomonas maltophilia is an uncommon but emerging nosocomial pathogen that is usually resistant to widely used antibiotics (71). LHCG-binding sites were found in X. maltophilia (68). Because these lack complete functional units, some authors have hesitated to call them receptors. While both human LH and hCG bind to LHCG low-affinity binding sites, only hCG (not human LH) can bind to LHCG high-affinity binding sites. Other glycoproteins such as LH, FSH, and TSH do not bind to them (61, 68). Hormone binding to high-affinity LHCG-binding sites is known to stimulate cell proliferation and changes in cell morphology (72). These changes are stimulated by hCG, hLH and Xanthomonas CG (72). The entire gene of chorionic gonadotropin has been isolated from X. maltophilia (Xanthomonas CG), was completely sequenced and showed homology to human CG and human LHCG receptor (46, 68)(See **Table 2**). The molecular weight of fungal CG is greater than the microbial CG which is greater than mammalian CGs (28, 46, 47) (See **Tables 2**, **3** and **Figure 5**).

**Table 2:** Homology between bacterial CG and human CG.

	Homology to human	Location of homology
**Xanthomonas CG**	46% homology to human CG	The body of β-subunit in amino acids 1761-1994 and 25-aa region of the C-terminus
**LHCG binding site in Xanthomonas**	73% homology to LHCG receptor	The human LHCG receptor

The entire gene of chorionic gonadotropin isolated from Xanthomonas was completely sequenced and showed 46% homology in the body of hCG β-subunit in amino acid 1761-1994 and in the 25-aa region of the carboxyl-terminal of hCG (42). The DNA sequence of the LHCG-binding site was even more similar to the human receptor, with 73% homology (68).

## Chorionic Gonadotropin as an Anti-Inflammatory Agent

### Pregnancy and hCG

The main endocrine roles of hCG are to maintain pregnancy during the first trimester, prevent corpus luteum regression, and support ovarian progesterone secretion (40). Other well-known roles of this hormone are to promote angiogenesis within the uterus, preserve progesterone production by the corpus luteum, maintain myometrial quiescence, and maintain local immune tolerance (40, 74). In addition to these roles associated with pregnancy, hCG shows a wide range of significant anti-inflammatory effects.

**Figure 6:**
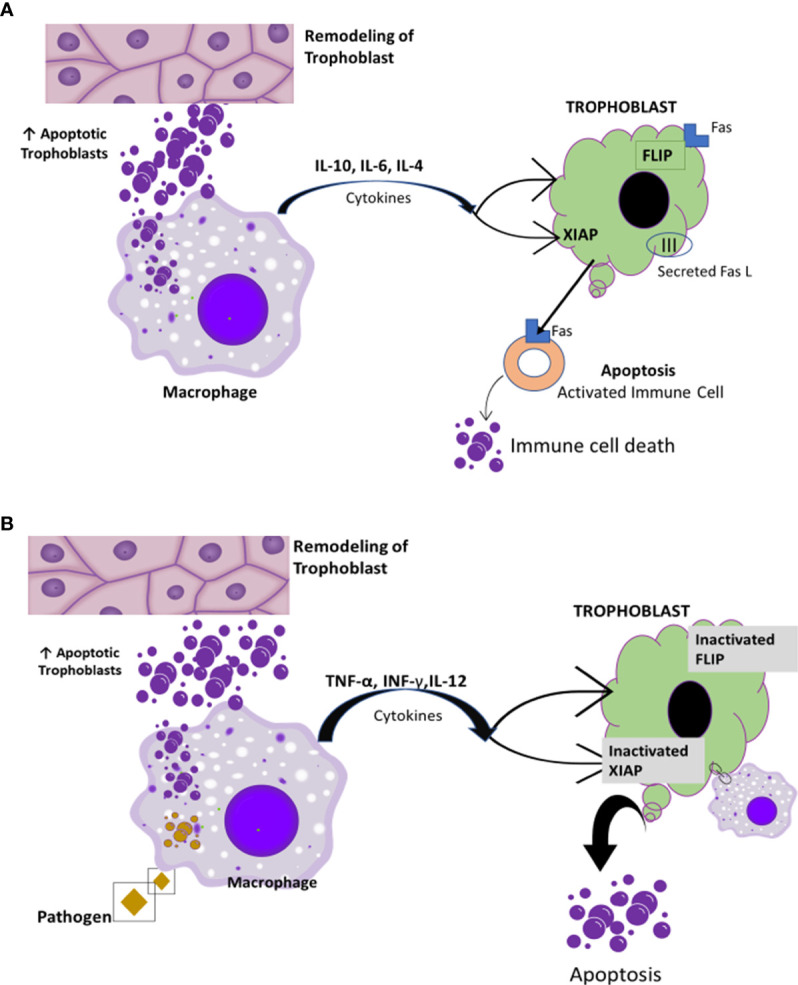
Macrophage clearance effect of apoptotic cells. [Reproduced from (78)]. **(A)** Macrophages engulf apoptotic cells. This clearance induces the expression of anti-inflammatory cytokines such IL-4, IL-5, and IL-10 along with immunological tolerance and protection of trophoblast survival (78, 80). **(B)** Inefficient clearance and high level of apoptotic bodies induce pro-inflammatory cytokine predominance. This possibly results in trophoblast resistance to apoptosis mediated by Fas and the maternal immune system (78).

Recall that the immune system is suppressed in pregnancy; this is accompanied by an increase in vulnerability to infections (75). Pregnancy is considered to be a controlled state of inflammation (76). In the early stages of pregnancy, inflammation is present locally at the site of implantation. In the later stages of pregnancy, inflammation extends systemically *via* the maternal circulation (77). The systemic inflammatory response in normal pregnancy is very similar to findings in patients with sepsis i.e., leukocytosis, increased monocyte priming, increased phagocytic activities, and increased production of pro-inflammatory cytokines such as interleukin 2 (IL-2), interleukin 6 (IL-6), and TNF-α (76, 77). Remarkably, these inflammatory changes do not appear to harm the mother or the fetus (76). One of the protective agents is hCG (77), which activates macrophages directly, especially their innate immune functions. Macrophages produce oxygen radicals for the mother’s defense against microorganisms and enhance phagocytic activities to clear apoptotic cells that are essential for resolution of local inflammation (77). In pregnancy, apoptosis is important for tissue remodeling and placental invasion during implantation (78, 79). Fas and Fas ligand (FasL) are involved in regulation of cell death (76). FasL mediates apoptotic processes to enhance placental invasion during implantation (79). Macrophages engulf the apoptotic cells, thereby preventing or retarding the potential pro-inflammatory actions generated by apoptotic cells (78) (**Figure 6**).

hCG, with interferon gamma (IFN-γ)-primed macrophages, significantly increases nitric oxide (NO) production and reactive oxygen species (ROS) that are cytotoxic for microorganisms including fungi, protozoa, bacteria and viruses. These free radicals offer crucial protection against microorganisms potentially dangerous for both mother and fetus (81). These functions of macrophages are vital to the maintenance of pregnancy and important to understand the “harmless” controlled state of sterile inflammation in pregnancy as well as hCG’s therapeutic benefits in acute inflammation (77).

In early stages of pregnancy, hCG contributes to maternal-fetal tolerance by increasing the migration of regulatory T cells (Tregs) into the maternal-fetal interface, thereby increasing Tregs in the lymphatic organs and circulation. Tregs promote activities *in vivo* that increase the production of anti-inflammatory cytokines such as IL-10 and of TGF-β (82, 83), which in turn dampen TNF-α, a pro-inflammatory cytokine. hCG also enhances a tolerogenic phenotype of bone marrow-derived dendritic cells (DCs) (74, 82, 84, 85). Zhou et al. have confirmed that for successful *in vitro* fertilization(IVF) Treg expansion and successful pregnancy are positively associated with increasing numbers of Tregs in the peripheral blood (86).

**Figure 7:**
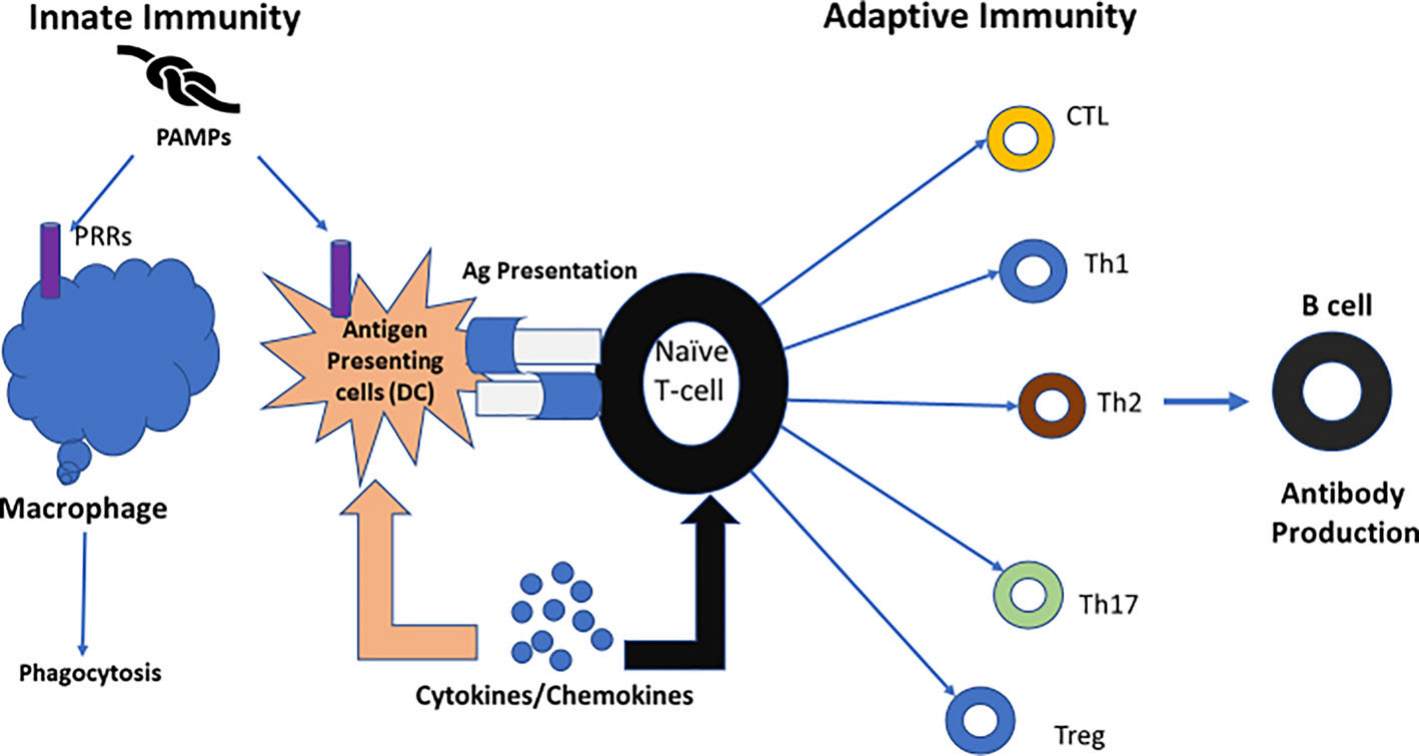
The role of dendritic cells; linking innate immunity and adaptive immunity. [Reproduced from (89)]. One key role of dendritic cells is linking innate immunity to adaptive immunity. When confronted with microbial antigens, dendritic cells (DCs) mature and migrate into draining lymph nodes where they present antigens to naïve T lymphocytes. Different pathogens trigger distinct dendritic cell maturation profiles and lead to the polarization of different T-cell subsets. Then, the adaptive immune response is modulated to match the nature of the pathogen (89). Ag, antigen; CTL, cytotoxic T cell; DC, dendritic cell; PRRs, Pattern recognition receptors; PAMPs, Pathogen associated molecular patterns. Figure from Intechopen (89) permissible to reuse under a CC-BY 4.0 license.

Macrophages and dendritic cells are involved in the innate immune response. Although macrophages are stimulated by foreign entities, they are not able to initiate a primary immune response (87). Dendritic cells, acting as antigen-presenting cells (APCs), can initiate a primary immune response by stimulating naive T cells (88). This is the interface between the innate and adaptive immune responses promoted by dendritic cells (87, 89). hCG regulates dendritic cell function by enhancing maternal-fetal immune tolerance (85) (**Figure 7**).

### Tempering Inflammation With hCG

**Figure 8:**
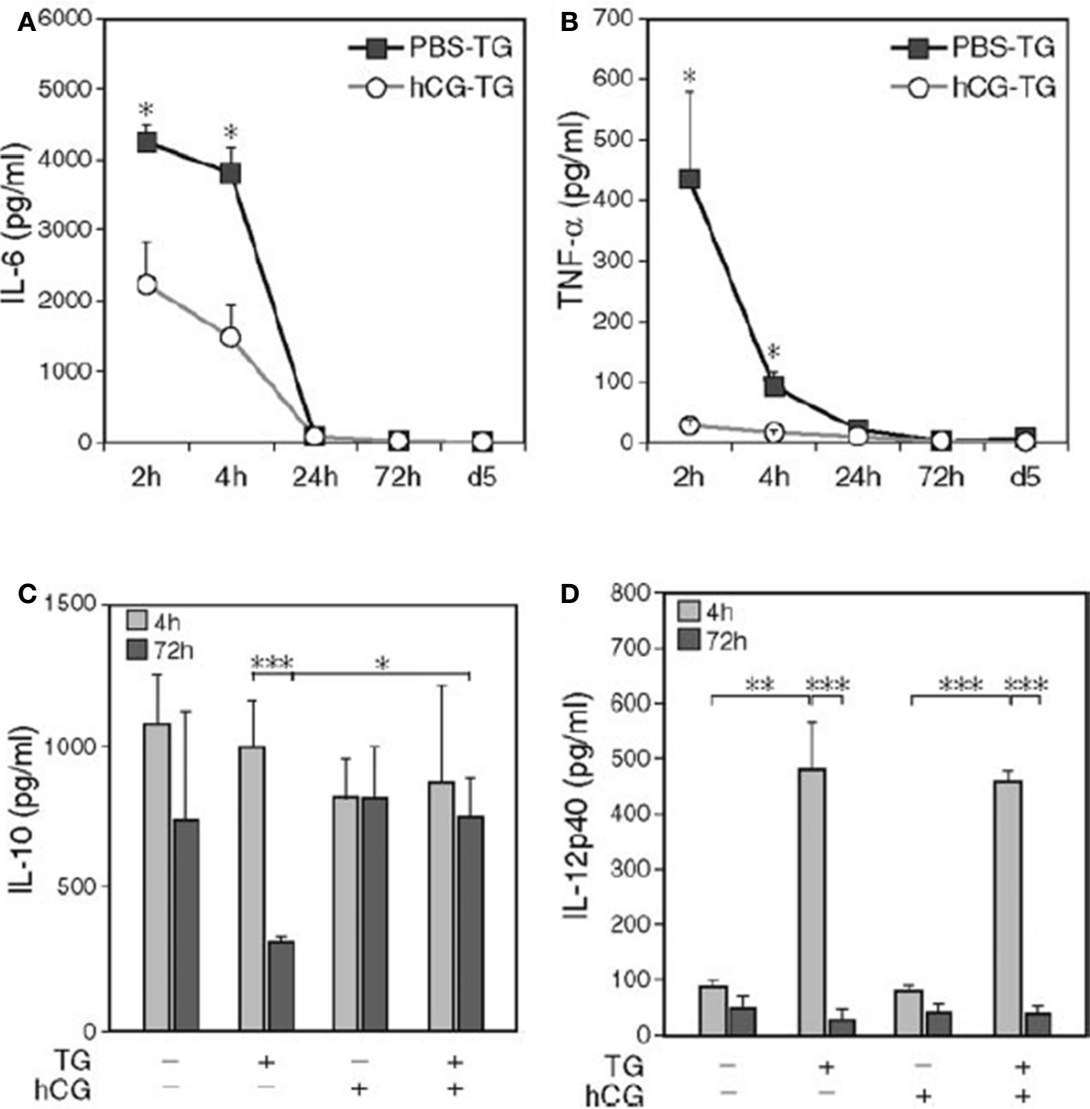
hCG pre-treatment reduces the level of IL-6 and TNF-α in peritoneal lavage fluid (77). C57BL/6 mice were injected intraperitoneally with hCG or PBS. One hour later, TG was intraperitoneally injected after 2 h, 4 h, 24 h (Day 1), and 72 h (Day 3). On Day 5, mice were sacrificed, and peritoneal lavage fluid was collected, and cytokine measured. Upon hCG pretreatment of TG-induced peritonitis, decreased IL-6 **(A)** and TNF-α levels **(B)** at 2 h and 4 h and a higher IL-10 level **(C)** at 72 h were observed, whereas IL-12p40 remained unchanged **(D)**; n = 15. Kinetic data depicted are from a separate representative experiment with five mice per group **(A, B)**. *P < 0.05; **P < 0.01; ***P < 0.001.

Many studies have demonstrated the anti-inflammatory influences of hCG. For example, Wan et al, found that in C57BL/6 female mice with thioglycolate (TG)-induced peritonitis, hCG pre-treatment diminished inflammation-induced cell death and decreased pro-inflammatory cytokine levels including IL-6, TNF-α, PTX3, CCL3, and CCL5 (77) (**Figure 8**).

BALB/c mice with acute liver injury induced by anti-Fas antibody (Jo 2) and agonistic CD95-antibodieshad significantly reduced levels of alanine aminotransferase (ALT) and aspartate aminotransferase (AST) when also treated with hCG. Mice treated with hCG also showed less CD4^+^ T cell infiltration and fewer apoptotic hepatocytes, confirming the effectiveness of hCG as an anti-inflammatory agent (90).

Signs of rheumatoid arthritis (RA) were induced in rats by the injection of streptococcal cell wall (SCW). Non-pregnant rats showed joint swelling, pro-inflammatory cell infiltration, increases in TNF-α, IL-6, IL-1β, NO, and inducible nitric oxide synthase (iNOS). hCG administration reduced signs of arthritis (91).

### Anti-Inflammatory Effects in Humans

Reduced symptoms of autoimmune diseases including rheumatoid arthritis (RA) and Sjogren syndrome (SS) have been reported during pregnancy (92, 93). Hazes et al. found a decrease in risk of RA among women who have been pregnant compared to nulligravid women [odds ratio was 0.49 (0.27-0.91)]. An early first pregnancy is associated with lower risk of RA (92). The contribution of hCG to this amelioration was suggested by several investigators (92, 93).

## Synthetic Peptides Related to hCG: LQGV, AQGV, and LAGV

Van den Berg et al. concluded that the anti-inflammatory effects of hCG are derived from peptides located in the hCG β-subunit such as LQGV, AQGV, and LAGV (94–97). Using rats with hemorrhagic shock they demonstrated the anti-inflammatory effects of these peptides. Hypotension (a mean arterial pressure of 40 mmHg) was maintained for 60 minutes. Groups of rats received either 5 mg/kg of LQGV, AQGV, LAGV, or normal saline (94). Administration of LQGV, AQGV, and LAGV prevented the release of IL-6 and TNF-α into the plasma and attenuated the rise of IL-6 and TNF-α mRNA transcript levels in the liver. LQGV treatment also attenuated the accumulation of neutrophils in the liver and the rise of aspartate aminotransferase (AST) and lactate dehydrogenase (LDH) levels while AQGV and LAGV treatment did not (94, 96).

### AQGV

Khan and his colleagues studied AQGV, an oligopeptide related to hCG-β, as an anti-inflammatory agent (97). They found that AQGV prevented mortality in mice with induced renal ischemia-reperfusion injury more effectively than other oligopeptides related to hCG-β by lowering neutrophil influx to the kidney, decreasing apoptosis, reducing proinflammatory cytokines such as TNF-α, INF-γ, IL-6 and IL-10, and increasing tubular epithelial cell proliferation (97) (**Table 4**).

**Table 4:** Effects of various oligopeptides (5 mg/kg) related to hCG-β on the survival rate of mice subjected to ischemia-reperfusion damage.

Treatment	Survival rate	
	24 h	72 h
**PBS (control)**	90%	50%
**AQGV**	100%	100%
**LQGV**	100%	80%
**LAGV**	90%	90%

[Modified from (97)].

### LQGV

A number of groups studied LQGV, another hCG-β related tetrapeptide, as a treatment for sepsis (94, 96, 98–100). The LQGV, leucine-glutamine-glycine-valine is present in loop 2 of the hCG-β subunit (96). Khan’s group found that the LQGV peptide showed a protective effect in mice with lethal LPS-induced septic shock and in rhesus monkeys with E. coli-induced septic shock (99). Following an injection of a lethal dose of LPS or E. coli to induce septic shock, mice and monkeys received LQGV or phosphate-buffered saline (PBS). The mice and monkeys that received LQGV demonstrated significantly improved hemodynamic parameters, improved sickness scores, and higher survival rates (99). LQGV treatment also showed anti-inflammatory effects on mice with CLP-induced sepsis. Van den Berg’s group induced sepsis in C57BL/6 mice with CLP and administered either LQGV or PBS as control to assess the anti-inflammatory effects of LQGV. Results demonstrated that LQGV treatment increased the survival rate up to 50% from 20% during acute phase of sepsis. LQGV treatment also decreased CLP-induced systemic cytokines (96).

## Conclusion

hCG is a major pregnancy hormone that belongs to the glycoprotein family. The well-known functions of hCG are related to pregnancy, such as the maintenance of the corpus luteum and angiogenesis of uterine vasculature. hCG is used in infertility treatment, prevention of postmenopausal symptoms and induction of testosterone production in hypogonadal men. Peptides similar to this hormone have been detected in microorganisms such as viruses, bacteria, protozoa, and fungi. The study of hCG recently has been expanded beyond its role as a pregnancy hormone to include studies demonstrating anti-inflammatory capabilities.

A number of pre-clinical and clinical studies have clearly demonstrated that the β-subunit of hCG and its related oligopeptides have anti-inflammatory properties. hCG and its related peptides show promise in the treatment of inflammatory diseases and sepsis to mitigate organ failure and reduce mortality. Further clinical studies are warranted to establish its role as an anti-inflammatory agent, alone and in concert with other anti-inflammatory agents.

## Author Contributions

All authors listed have made a substantial, direct, and intellectual contribution to the work, and approved it for publication. Also note that SYK, SFM, SP, NM and JR substantially contributed to the conception and design of the article and interpretation of the relevant literature. BLu, DL, CNM, HY and JR added critical intellectual content to the manuscript and can be considered experts on the topic. All authors including MA, BLo and SAK provided critical feedback and helped shape the research and analysis.

## Funding

The authors declare that this study received philanthropic funding from Alan and Tatyana Forman through Altronix Inc. The funder was not involved in the study design, collection, analysis, interpretation of data, the writing of this article or the decision to submit it for publication.

## Conflict of Interest

The authors declare that the research was conducted in the absence of any commercial or financial relationships that could be construed as a potential conflict of interest.

## Publisher’s Note

All claims expressed in this article are solely those of the authors and do not necessarily represent those of their affiliated organizations, or those of the publisher, the editors and the reviewers. Any product that may be evaluated in this article, or claim that may be made by its manufacturer, is not guaranteed or endorsed by the publisher.

## References

[1] Vincent JL Opal SM Marshall JC Tracey KJ. Sepsis Definitions: Time for Change. Lancet (London England) (2013) 381(9868):774–5. doi: 10.1016/S0140-6736(12)61815-7 PMC4535310 23472921

[2] Rello J Valenzuela-Sánchez F Ruiz-Rodriguez M Moyano S. Sepsis: A Review of Advances in Management. Adv Ther (2017) 34(11):2393–411. doi: 10.1007/s12325-017-0622-8 PMC5702377 29022217

[3] Napolitano LM. Sepsis 2018: Definitions and Guideline Changes. Surg Infect (Larchmt) (2018) 19(2):117–25. doi: 10.1089/sur.2017.278 29447109

[4] WHO. Sepsis. Geneva, Switzerland: World Health Organization (2018). Available at: https://www.who.int/news-room/fact-sheets/detail/sepsis.

[5] NIH. Sepsis. Bethesda, Maryland, USA: National Institute of Health (2019). Available at: https://www.nigms.nih.gov/education/pages/factsheet_sepsis.aspx.

[6] Rubens M Saxena A Ramamoorthy V Das S Khera R Hong J. Increasing Sepsis Rates in the United States: Results From National Inpatient Sample, 2005 to 2014. J Intensive Care Med (2020) 35(9):858–68. doi: 10.1177/0885066618794136 30175649

[7] Ward PA. New Approaches to the Study of Sepsis. EMBO Mol Med (2012) 4(12):1234–43. doi: 10.1002/emmm.201201375 PMC3531600 23208733

[8] Tavakoli A Carannante A. Nursing Care of Oncology Patients With Sepsis. Semin Oncol Nurs (2021) 37(2):151130. doi: 10.1016/j.soncn.2021.151130 33722431

[9] Gipponi M Sciutto C Accornero L Bonassi S Raso C Vignolo C. Assessing Modifications of the Intestinal Bacterial Flora in Patients on Long-Term Oral Treatment With Bacampicillin or Amoxicillin: A Random Study. Chemioterapia (1985) 4(3):214–7. 4028281

[10] Cole LA. New Discoveries on the Biology and Detection of Human Chorionic Gonadotropin. Reprod Biol Endocrinol (2009) 7:8. doi: 10.1186/1477-7827-7-8 19171054 PMC2649930

[11] Hirose T. Exogenous Stimulation of Corpus Luteum Formation in the Rabbit: Influence of Extracts of Human Placenta, Decidua, Fetus, Hydatid Mole, and Corpus Luteum on the Rabbit Gonad. Japan Soc Obstet Gynecol (1920) 16:1055.

[12] Lunenfeld B. Historical Perspectives in Gonadotrophin Therapy. Hum Reprod Update (2004) 10(6):453–67. doi: 10.1093/humupd/dmh044 15388674

[13] Lunenfeld B Bilger W Longobardi S Alam V D’Hooghe T Sunkara SK. The Development of Gonadotropins for Clinical Use in the Treatment of Infertility. Front Endocrinol (2019) 10:429. doi: 10.3389/fendo.2019.00429 PMC6616070 31333582

[14] Practice Committee of American Society for Reproductive Medicine, Birmingham, Alabama. Gonadotropin Preparations: Past, Present, and Future Perspectives. Fertil Steril (2008) 90(5 Suppl):S13–20. doi: 10.1016/j.fertnstert.2008.08.031 19007609

[15] Ettinger GH Smith GL McHenry EW. The Diagnosis of Pregnancy With the Aschheim-Zondek Test. Can Med Assoc J (1931) 24(4):491–5. PMC382392 20318243

[16] Borth R Lunenfeld B Stamm O De Watteville. Gonadotrophin and Steroid Excretion in a Case of Twin Pregnancy. Acta Obstet Gynecol Scand (1959) 38:417–23. doi: 10.3109/00016345909153938 13802985

[17] Lunenfeld B. Induction of Ovulation in Pituitary Amenorrhea by Combined Treatment of Menopausal Urinary Gonadotropins and Chorionic Gonadotropins. CR Soc Franc Gynecol (1962) 32(5):346.

[18] Wide L Gemzell CA. An Immunological Pregnancy Test. Acta Endocrinol (1960) 35:261–7. doi: 10.1530/acta.0.xxxv0261 13785019

[19] Isojima S Koyama K Tanaka C Adachi H. Radioimmunoassay of Human Urinary Chorionic Gonadotropin (HCG) and Luteinizing Hormone (LH). Nihon Naibunpi Gakkai Zasshi (1968) 43(11):1097–108. doi: 10.1507/endocrine1927.43.11_1097 4173119

[20] Best CH McHenry EW. The Friedman Modification of the Aschheim-Zondek Test for the Diagnosis of Pregnancy. Can Med Assoc J (1933) 28(6):599–600. 20319126 PMC402870

[21] Cole LA. History and Introduction to Human Chorionic Gonadotropin (hCG): One Name for at Least Three Independent Molecules. Hum Chorionic Gonadotropin (2010), 13–22. doi: 10.1016/B978-0-12-384907-6.00002-5

[22] Lunenfeld B. Gonadotropin Stimulation: Past, Present and Future. Reprod Med Biol (2011) 11(1):11–25. doi: 10.1007/s12522-011-0097-2 29699102 PMC5906949

[23] Gurin S Bachman C Wilson DW. The Gonadotropic Hormone of Urine of Pregnancy: II. Chemical Studies of Preparations Having High Biological Activity. J Biol Chem (1940) 133(2):467–76. doi: 10.1016/S0021-9258(18)73326-7

[24] Katzman PA Godfrid M Cain CK Doisy EA. The Preparation of Chorionic Gonadotropin by Chromatographic Adsorption. J Biol Chem (1943) 148(3):501–7. doi: 10.1016/S0021-9258(18)72248-5

[25] Esteves SC. Efficacy, Efficiency and Effectiveness of Gonadotropin Therapy for Infertility Treatment. MEDICALEXPRESS (2015) 2(3):M150302. doi: 10.5935/MedicalExpress.2015.03.02

[26] Mullen MP Cooke DJ Crow MA. Structural and Functional Roles of FSH and LH as Glycoproteins Regulating Reproduction in Mammalian Species. In: Gonadotropin, Jorge Vizcarra. London, United Kingdom: IntechOpen (2013). doi: 10.5772/48681

[27] Solarski M Rotondo F Syro LV Cusimano MD Kovacs K. Alpha Subunit in Clinically Non-Functioning Pituitary Adenomas: An Immunohistochemical Study. Pathol Res Pract (2017) 213(9):1130–3. doi: 10.1016/j.prp.2017.07.010 28780083

[28] Fournier T Guibourdenche J Evain-Brion D. Review: hCGs: Different Sources of Production, Different Glycoforms and Functions. Placenta (2015) 36 Suppl;1:S60–5. doi: 10.1016/j.placenta.2015.02.002 25707740

[29] Cole LA. Structures of Free α- and β-Subunits. 1st ed. Amsterdam, Netherlands: Elsevier Inc (2010). doi: 10.1016/B978-0-12-384907-6.00007-4

[30] Cole LA. Structures of HCG Free α-Subunit and Free β-Subunit. Amsterdam, Netherlands: Elsevier Inc (2015). doi: 10.1016/b978-0-12-800749-5.00006-7

[31] Lustbader JW Pollak S Lobel L Trakht I Homans S Brown JM. Three-Dimensional Structures of Gonadotropins. Mol Cell Endocrinol (1996) 125(1-2):21–31. doi: 10.1016/S0303-7207(96)03952-4 9027340

[32] Anderson RC Newton CL Anderson RA Millar RP. Gonadotropins and Their Analogs: Current and Potential Clinical Applications. Endocr Rev (2018) 39(6):911–37. doi: 10.1210/er.2018-00052 29982442

[33] Ascoli M Segaloff DL. On the Structure of the Luteinizing Hormone/Chorionic Gonadotropin Receptor. Endocr Rev (1989) 10(1):27–44. doi: 10.1210/edrv-10-1-27 2666109

[34] Cooke DJ Crowe MA Roche JF Headon DR. Gonadotrophin Heterogeneity and Its Role in Farm Animal Reproduction. AnimReprod Sci (1996) 41(2):77–99. doi: 10.1016/0378-4320(95)01449-7

[35] Closset J Hennen G Lequin RM. Human Luteinizing Hormone: The Amino Acid Sequence of the β Subunit. ObstetGynecolSurv (1973) 28(10):736–7. doi: 10.1097/00006254-197310000-00018 4719207

[36] Morgan F Birken S Canfield R. The Amino Acid Sequence of Human Chorionic Gonadotropin. The Alpha-Subunit and the Beta-Subunit. J Biol Chem (1975) 250(13):5247–58. doi: 10.1016/S0021-9258(19)41303-3 1150658

[37] Kobata A Takeuchi M. Structure, Pathology and Function of the N-Linked Sugar Chains of Human Chorionic Gonadotropin. BiochimBiophys Acta - Mol Basis Dis (1999) 1455(2–3):315–26. doi: 10.1016/S0925-4439(99)00060-5 10571021

[38] Nwabuobi C Arlier S Schatz F Guzeloglu-Kayisli O Lockwood CJ Kayisli UA. hCG: Biological Functions and Clinical Applications. Int J Mol Sci (2017) 18(10):1–15. doi: 10.3390/ijms18102037 PMC5666719 28937611

[39] Costa MA. The Endocrine Function of Human Placenta: An Overview. Reprod BioMed Online (2016) 32(1):14–43. doi: 10.1016/j.rbmo.2015.10.005 26615903

[40] Fournier T. Human Chorionic Gonadotropin: Different Glycoforms and Biological Activity Depending on Its Source of Production. Ann Endocrinol (Paris) (2016) 77(2):75–81. doi: 10.1016/j.ando.2016.04.012 27177499

[41] Grover S Woodward SR Odell WD. A Bacterial Protein has Homology With Human Chorionic Gonadotropin (hCG). BiochemBiophys Res Commun (1993) 193(3):841–7. doi: 10.1006/bbrc.1993.1702 8323559

[42] Fujiki Y Rathnam P Saxena BB. Amino Acid Sequence of the β-Subunit of the Follicle-Stimulating Hormone From Equine Pituitary Glands. J Biol Chem (1978) 253(15):5363–8. doi: 10.1016/S0021-9258(17)30379-4 670202

[43] Bischof P Islami D. Sexual Hormones. Geneva, Switzerland: Geneva Foundation for Medical Education and Research (2017). Available at: https://www.gfmer.ch/Endo/Lectures_08/sexual_hormones.htm.

[44] Szkudlinski MW Fremont V Ronin C Weintraub BD. Thyroid-Stimulating Hormone and Thyroid-Stimulating Hormone Receptor Structure-Function Relationships. Physiol Rev (2002) 82(2):473–502. doi: 10.1152/physrev.00031.2001 11917095

[45] Luteinizing Hormone Beta Protein. Fitzgerald (2019). Available at: https://www.fitzgerald-fii.com/luteinizing-hormone-beta-protein-30-al35.html.

[46] Grover S Woodward SR Odell WD. Complete Sequence of the Gene Encoding a Chorionic Gonadotropin-Like Protein From Xanthomonas Maltophilia. Gene (1995) 156(1):75–8. doi: 10.1016/0378-1119(95)00056-C 7537705

[47] Caticha O Odell WD. Characterization and Purification of the Chorionic Gonadotrofin-Like Protein Binding Site in Candida Albicans. Endocr Res (1994) 20(1):1–19. doi: 10.1080/07435809409035852 8168460

[48] Odell WD Griffin J Grover S Carrell DT. Human Chorionic Gonadotropin-Like Proteins: Secretion in Nonpregnant Humans and Production by Bacteria. Trans Am Clin Climatol Assoc (1992) 103:235–54. PMC2376693 1413384

[49] Grover S Woodward SR Caticha O Carrell DT Odell WD. Partial Nucleotide Sequence of the Xanthomonas Maltophilia Chorionic Gonadotropin-Like Receptor. BiochemBiophys Res Commun (1993) 190(2):371–6. doi: 10.1006/bbrc.1993.1057 8427582

[50] Cole LA. Biological Functions of hCG and hCG-Related Molecules. Reprod Biol Endocrinol (2010) 8:1–14. doi: 10.1186/1477-7827-8-102 20735820 PMC2936313

[51] Backus BT Affronti LF. Tumor-Associated Bacteria Capable of Producing a Human Choriogonadotropin-Like Substance. Infect Immun (1981) 32(3):1211–5. doi: 10.1128/iai.32.3.1211-1215.1981 PMC351581 7019090

[52] Acevedo HF Slifkin M Pouchet GR Pardo M. Immunohistochemical Localization of a Choriogonadotropin-Like Protein in Bacteria Isolated From Cancer Patients. Cancer (1978) 41(4):1217–29. doi: 10.1002/1097-0142(197804)41:4<1217::aid-cncr2820410401>3.0.co;2-a 76504

[53] Acevedo HF Koide SS Slifkin M Maruo T Campbell-Acevedo EA. Choriogonadotropin-Like Antigen in a Strain of Streptococcus Faecalis and a Strain of Staphylococcus Simulans: Detection, Identification, and Characterization. Infect Immun (1981) 31(1):487–94. doi: 10.1128/IAI.31.1.487-494.1981 PMC351808 6783540

[54] Acevedo HF Slifkin M Pouchet-Melvin GR Campbell-Acevedo EA. Choriogonadotropin-Like Antigen in an Anaerobic Bacterium, Eubacterium Lentum, Isolated From a Rectal Tumor. Infect Immun (1979) 24(3):920–4. doi: 10.1128/IAI.24.3.920-924.1979 PMC414395 468380

[55] Iwasa Y Yonemitsu K Matsui K. Calmodulin-Like Activity in the Soluble Fraction of Escherichia Coli. BiochemBiophys Res Commun (1981) 98(3):656–60. doi: 10.1016/0006-291x(81)91164-5 6261747

[56] LeRoith D Shiloach J Roth J Lesniak MA. Insulin or a Closely Related Molecule is Native to Escherichia Coli. J Biol Chem (1981) 256(13):6533–6. doi: 10.1016/S0021-9258(19)69020-4 7016870

[57] Macchia V Bates RW Pastan I. The Purification and Properties of a Thyroid-Stimulating Factor Isolated From Clostridium Perfringens. J Biol Chem (1967) 242(16):3726–30. doi: 10.1016/S0021-9258(18)95869-2 4292226

[58] Bhatnagar YM Carraway R. Bacterial Peptides With C-Terminal Similarities to Bovine Neurotensin. Peptides (1981) 2(1):51–9. doi: 10.1016/S0196-9781(81)80011-3 7017622

[59] Qiang X Liotta AS Shiloach J Gutierrez JC Wang H Ochani. New Melanocortin-Like Peptide of E. Coli can Suppress Inflammation *via* the Mammalian Melanocortin-1 Receptor (MC1R): Possible Endocrine-Like Function for Microbes of the Gut. NPJ Biofilms Microbiomes (2017) 3(1):1–10. doi: 10.1038/s41522-017-0039-9 29152323 PMC5684143

[60] Bramley TA Menzies GS Williams RJ Adams DJ Kinsman OS. Specific, High-Affinity Binding Sites for Human Luteinizing Hormone (hLH) and Human Chorionic Gonadotrophin (hCG) in Candida Species. BiochemBiophys Res Commun (1990) 167(3):1050–6. doi: 10.1016/0006-291x(90)90629-2 2108673

[61] Carrell DT Odell WD. A Bacterial Binding Site Which Binds Human Chorionic Gonadotropin But Not Human Luteinizing Hormone. Endocr Res (1992) 18(1):51–8. doi: 10.3109/07435809209035928 1576977

[62] Domingue GJ Acevedo HF Powell JE Stevens VC. Antibodies to Bacterial Vaccines Demonstrating Specificity for Human Choriogonadotropin (hCG) and Immunochemical Detection of hCG-Like Factor in Subcellular Bacterial Fractions. Infect Immun (1986) 53(1):95–8. doi: 10.1128/IAI.53.1.95-98.1986 PMC260080 3721581

[63] Livingston AM. Some Cultural, Immunological, and Biochemical Properties of Progenitor Cryptocides. Trans N Y Acad Sci (1974) 36(6):569–82. doi: 10.1111/j.2164-0947.1974.tb01602.x 4530542

[64] Richert ND Ryan RJ. Specific Gonadotropin Binding to Pseudomonas Maltophilia. Proc Natl Acad Sci USA (1977) 74(3):878–82. doi: 10.1073/pnas.74.3.878 PMC430513 265583

[65] Roth J LeRoith D Lesniak MA de Pablo F Bassas L Collier E. Molecules of Intercellular Communication in Vertebrates, Invertebrates and Microbes: Do They Share Common Origins? Prog Brain Res (1986) 68(C):71–9. doi: 10.1016/S0079-6123(08)60231-9 3562852

[66] Roth J Leroith D Collier ES Watkinson A Lesniak MA. The Evolutionary Origins of Intercellular Communication and the Maginot Lines of the Mind. Ann N Y Acad Sci (1986) 463(1):1–11. doi: 10.1111/j.1749-6632.1986.tb21498.x 3013064

[67] Maruo T Cohen H Segal SJ Koide SS. Production of Choriogonadotropin-Like Factor by a Microrganism. Proc Natl Acad Sci USA (1979) 76(12):6622–6. doi: 10.1073/pnas.76.12.6622 PMC411919 230516

[68] Edwards JG Odell WD. Partial Characterization of Chorionic Gonadotropin-Like Binding Sites From the Bacteria Xanthomonas Maltophilia. Exp Biol Med (2003) 228(8):926–34. doi: 10.1177/153537020322800808 12968064

[69] Acevedo HF Pardo M Campbell-Acevedo E Domingue GJ. Human Choriogonadotropin-Like Material in Bacteria of Different Species: Electron Microscopy and Immunocytochemical Studies With Monoclonal and Polyclonal Antibodies. J Gen Microbiol (1987) 133(3):783–91. doi: 10.1099/00221287-133-3-783 3116165

[70] Acevedo HF Campbell-Acevedo E Kloos WE. Expression of Human Choriogonadotropin-Like Material in Coagulase-Negative Staphylococcus Species. Infect Immun (1985) 50(3):860–8. doi: 10.1128/IAI.50.3.860-868.1985 PMC261159 2415456

[71] Brooke JS. Stenotrophomonas Maltophilia: An Emerging Global Opportunistic Pathogen. Clin Microbiol Rev (2012) 25(1):2–41. doi: 10.1128/CMR.00019-11 22232370 PMC3255966

[72] Carrell DT Elizabeth Hammond M Odell D. Evidence for an Autocrine/Paracrine Function of Chorionic Gonadotropin in Xanthomonas Maltophilia. Endocrinology (1993) 132(3):1085–9. doi: 10.1210/endo.132.3.7679968 7679968

[73] LeãoRde B Esteves SC. Gonadotropin Therapy in Assisted Reproduction: An Evolutionary Perspective From Biologics to Biotech. Clinics (Sao Paulo) (2014) 69(4):279–93. doi: 10.6061/clinics/2014(04)10 PMC3971356 24714837

[74] Furcron AE Romero R Mial TN Balancio A Panaitescu B Hassan SS. Human Chorionic Gonadotropin Has Anti-Inflammatory Effects at the Maternal-Fetal Interface and Prevents Endotoxin-Induced Preterm Birth, But Causes Dystocia and Fetal Compromise in Mice1. Biol Reprod (2016) 94(6):1–13. doi: 10.1095/biolreprod.116.139345 PMC4946806 27146032

[75] Mor G Cardenas I Abrahams V Guller S. Inflammation and Pregnancy: The Role of the Immune System at the Implantation Site. Ann N Y Acad Sci (2011) 1221(1):80–7. doi: 10.1111/j.1749-6632.2010.05938.x.Inflammation PMC3078586 21401634

[76] Sargent IL Borzychowski AM Redman CWG. NK Cells and Human Pregnancy – an Inflammatory View. Trends Immunol (2006) 27(9):399–404. doi: 10.1016/j.it.2006.06.009 16843067

[77] Wan H Coppens JMC van Helden-Meeuwsen CG Leenen PJ van Rooijen N Khan NA. Chorionic Gonadotropin Alleviates Thioglycollate-Induced Peritonitis by Affecting Macrophage Function. J Leukoc Biol (2009) 86(2):361–70. doi: 10.1189/jlb.0208126 19414540

[78] Mor G Abrahams VM. Potential Role of Macrophages as Immunoregulators of Pregnancy. Reprod Biol Endocrinol (2003) 1:1–8. doi: 10.1186/1477-7827-1-119 PMC305335 14651752

[79] Uckan D. Trophoblasts Express Fas Ligand: A Proposed Mechanism for Immune Privilege in Placenta and Maternal Invasion. Mol Hum Reprod (1997) 3(8):655–62. doi: 10.1093/molehr/3.8.655 9294848

[80] Opal SM DePalo VA. Anti-Inflammatory Cytokines. Chest (2000) 117(4):1162–72. doi: 10.1378/chest.117.4.1162 10767254

[81] Wan H Versnel MA Cheung WY Leenen PJ Khan NA Benner R. Chorionic Gonadotropin Can Enhance Innate Immunity by Stimulating Macrophage Function. J Leukoc Biol (2007) 82(4):926–33. doi: 10.1189/jlb.0207092 17626151

[82] Schumacher A Brachwitz N Sohr S Engeland K Langwisch S Dolaptchieva M. Human Chorionic Gonadotropin Attracts Regulatory T Cells Into the Fetal-Maternal Interface During Early Human Pregnancy. J Immunol (2009) 182(9):5488–97. doi: 10.4049/jimmunol.0803177 19380797

[83] Schumacher A Heinze K Witte J Poloski E Linzke N Woidacki K. Human Chorionic Gonadotropin as a Central Regulator of Pregnancy Immune Tolerance. J Immunol (2013) 190(6):2650–8. doi: 10.4049/jimmunol.1202698 23396945

[84] Rolle L Memarzadeh Tehran M Morell-García A Raeva Y Schumacher A Hartig R. Cutting Edge: IL-10-Producing Regulatory B Cells in Early Human Pregnancy. Am J Reprod Immunol (2013) 70(6):448–53. doi: 10.1111/aji.12157 24118333

[85] Wan H Versnel MA Leijten LME van Helden-Meeuwsen CG Fekkes D Leenen PJ. Chorionic Gonadotropin Induces Dendritic Cells to Express a Tolerogenic Phenotype. J Leukoc Biol (2008) 83(4):894–901. doi: 10.1189/jlb.0407258 18171698

[86] Zhou J Wang Z Zhao X Wang J Sun H Hu Y. An Increase of Treg Cells in the Peripheral Blood Is Associated With a Better *In Vitro* Fertilization Treatment Outcome. Am J Reprod Immunol (2012) 68(2):100–6. doi: 10.1111/j.1600-0897.2012.01153.x 22687138

[87] Clark GJ Angel N Kato M López JA MacDonald K Vuckovic S. The Role of Dendritic Cells in the Innate Immune System. Microbes Infect (2000) 2(3):257–72. doi: 10.1016/S1286-4579(00)00302-6 10758402

[88] Howard CJ Charleston B Stephens SA Sopp P Hope JC. The Role of Dendritic Cells in Shaping the Immune Response. Anim Heal Res Rev (2004) 5(2):191–5. doi: 10.1079/ahr200468 15984324

[89] Miguel B Celeste M Teresa M. Pathogen Strategies to Evade Innate Immune Response: A Signaling Point of View. Da Silva Xavier G, editor. London, United Kingdom: IntechOpen (2012). doi: 10.5772/37771

[90] Steinmetz C Kashyap A Zhivkova N Alizor H Ernst I Gottfried-Brand D. Activation of Silent Mating Type Information Regulation 2 Homolog 1 by Human Chorionic Gonadotropin Exerts a Therapeutic Effect on Hepatic Injury and Inflammation. Hepatology (2017) 65(6):2074–89. doi: 10.1002/hep.29072 28108987

[91] Rao CV. Potential Therapy for Rheumatoid Arthritis and Sjögren Syndrome With Human Chorionic Gonadotropin. Reprod Sci (2016) 23(5):566–71. doi: 10.1177/1933719115597765 26239386

[92] Hazes JMW Dijkmans BAC Vandenbroucke JP Vries RRPD Cats A. Pregnancy and the Risk of Developing Rheumatoid Arthritis. Arthritis Rheum (1990) 33(12):1770–5. doi: 10.1002/art.1780331203 2260999

[93] Wilder RL. Hormones, Pregnancy, and Autoimmune Diseases. Ann N Y Acad Sci (1998) 1(840):45–50. doi: 10.1111/j.1749-6632.1998.tb09547.x 9629235

[94] van den Berg HR Khan NA van der Zee M Bonthuis F IJzermans JN Dik WA. Synthetic Oligopeptides Related to the β-Subunit of Human Chorionic Gonadotropin Attenuate Inflammation and Liver Damage After (Trauma) Hemorrhagic Shock and Resuscitation. Shock (2009) 31(3):285–91. doi: 10.1097/SHK.0b013e31817fd62a 18654091

[95] Van Der Zee M Van Den Berg JW Van Holten-Neelen C Dik WA. The B-Human Chorionic Gonadotropin-Related Peptide LQGV Exerts Anti-Inflammatory Effects Through Activation of the Adrenal Gland and Glucocorticoid Receptor in C57BL/6 Mice. J Immunol (2007) 185(9):5066–73. doi: 10.4049/jimmunol.1001414 20926791

[96] van den Berg JW Dik WA van der Zee M Bonthuis F van Holten-Neelen C Dingjan GM. The β-Human Chorionic Gonadotropin-Related Peptide LQGV Reduces Mortality and Inflammation in a Murine Polymicrobial Sepsis Model. Crit Care Med (2011) 39(1):126–34. doi: 10.1097/CCM.0b013e3181fa3a93 20890188

[97] Khan NA Susa D Van Den Berg JW Huisman M Ameling MH van den Engel S. Amelioration of Renal Ischaemia-Reperfusion Injury by Synthetic Oligopeptides Related to Human Chorionicgonadotropin. Nephrol Dial Transplant (2009) 24(9):2701–8. doi: 10.1093/ndt/gfp369 19633318

[98] Van Der Zee M Van Den Berg JW Van Holten-Neelen C Dik WA. The B-Human Chorionic Gonadotropin-Related Peptide LQGV Exerts Anti-Inflammatory Effects Through Activation of the Adrenal Gland and Glucocorticoid Receptor in C57BL/6 Mice. J Immunol (2013) 185(9):5066–73. doi: 10.4049/jimmunol.1102538 20926791

[99] Khan NA Vierboom MP Van Holten - Neelen C Breedveld E Zuiderwijk-Sick E Khan A. Mitigation of Septic Shock in Mice and Rhesus Monkeys by Human Chorionic Gonadotrophin-Related Oligopeptides. Clin Exp Immunol (2010) 160(3):466–78. doi: 10.1111/j.1365-2249.2010.04112.x PMC2883119 20345979

[100] Zamorina SA Shirshev SV. Oligopeptides of Chorionic Gonadotropin β-Subunit in Induction of T Cell Differentiation Into Treg and Th17. Bull Exp Biol Med (2015) 160(1):72–5. doi: 10.1007/s10517-015-3101-8 26597689

